# Enhancing the Melting Process of Shell-and-Tube PCM Thermal Energy Storage Unit Using Modified Tube Design

**DOI:** 10.3390/nano12173078

**Published:** 2022-09-05

**Authors:** Aissa Abderrahmane, Naef A. A. Qasem, Abed Mourad, Mohammad Al-Khaleel, Zafar Said, Kamel Guedri, Obai Younis, Riadh Marzouki

**Affiliations:** 1 Laboratoire de Physique Quantique de la Matière et Modélisation Mathématique (LPQ3M), University Mustapha Stambouli of Mascara, Mascara 29000, Algeria; 2 Department of Aerospace Engineering and Interdisciplinary Research Center for Membranes and Water Security, King Fahd University of Petroleum & Minerals (KFUPM), Dhahran 31261, Saudi Arabia; 3 Department of Mathematics, Khalifa University, Abu Dhabi P.O. Box 127788, United Arab Emirates; 4 Department of Mathematics, Yarmouk University, Irbid 21163, Jordan; 5 Department of Sustainable and Renewable Energy Engineering, University of Sharjah, Sharjah P.O. Box 27272, United Arab Emirates; 6 U.S.-Pakistan Center for Advanced Studies in Energy (USPCAS-E), National University of Sciences and Technology (NUST), Islamabad 44000, Pakistan; 7 Mechanical Engineering Department, College of Engineering and Islamic Architecture, Umm Al-Qura University, Makkah 21955, Saudi Arabia; 8 Department of Mechanical Engineering, College of Engineering in Wadi Addwasir, Prince Sattam Bin Abdulaziz University, Al-Kharj 16278, Saudi Arabia; 9 Department of Mechanical Engineering, Faculty of Engineering, University of Khartoum, Khartoum 11111, Sudan; 10 Chemistry Department, College of Science, King Khalid University, Abha 61413, Saudi Arabia; 11 Chemistry Department, Faculty of Sciences of Sfax, University of Sfax, Sfax 3038, Tunisia

**Keywords:** shell-and-tube TES, nano-enhanced PCM, nanoparticles, fins, latent heat energy storage

## Abstract

Recently, phase change materials (PCMs) have gained great attention from engineers and researchers due to their exceptional properties for thermal energy storing, which would effectively aid in reducing carbon footprint and support the global transition of using renewable energy. The current research attempts to enhance the thermal performance of a shell-and-tube heat exchanger by means of using PCM and a modified tube design. The enthalpy–porosity method is employed for modelling the phase change. Paraffin wax is treated as PCM and poured within the annulus; the annulus comprises a circular shell and a fined wavy (trefoil-shaped) tube. In addition, copper nanoparticles are incorporated with the base PCM to enhance the thermal conductivity and melting rate. Effects of many factors, including nanoparticle concentration, the orientation of the interior wavy tube, and the fin length, were examined. Results obtained from the current model imply that Cu nanoparticles added to PCM materials improve thermal and melting properties while reducing entropy formation. The highest results (27% decrease in melting time) are obtained when a concentration of nanoparticles of 8% is used. Additionally, the fins’ location is critical because fins with 45° inclination could achieve a 50% expedition in the melting process.

## 1. Introduction

Energy storage will play an increasingly important role in the energy supply chain. The adoption of various thermal energy storage (TES) technologies is projected to increase, given that thermal energy accounts for a significant amount of overall energy consumption. In this framework, the past decade has seen a surge in research on latent heat thermal energy storage (LHTES) employing phase change materials (PCMs). A significant quantity of heat is absorbed/released during a material’s phase shift. This opens significant opportunities for LHTES and, therefore, for reducing the mismatch between heat supply and demand in terms of time, space, and rate. PCMs seem to be a potential strategy for energy saving and efficiency enhancement. When used in thermal systems, PCMs provide several benefits. Numerous studies have proven that this strategy has significant promise for successful renewable energy resource usage, waste heat recovery, and thermal management [1,2,3,4]. Additionally, the capital costs for electrochemical storage devices and LHTES were estimated to be USD 200–300 and USD 70–200 per kWh, respectively [5]. Further, it has been proved that LHTES could save the same amount of energy by using less volume during the same temperature change, resulting in better efficiency. PCMs are available at a range of melt points. This thermophysical feature is critical for determining how PCMs should be integrated into thermal systems. PCMs integrated into the building must be active at the human body thermal comfort temperature. Cooling an electrical device may be needed to operate at a significantly greater temperature, such as 58 degrees Celsius or more.

The phase change process and uses of PCMs have been extensively studied, both numerically and experimentally. Generally, an experimental prototype of a particular configuration is constructed and used to gather experimental data to validate the numerical model. Such a verified model may be used to investigate alternative configurations, regulate processes, or even optimize the design. Experiments that take time and money are often limited to a restricted range of operating circumstances, while the use of verified numerical models is quicker and more adaptable. The phase change process and heat transfer in a PCM are reliant on its thermophysical properties, the form of its container, and operating conditions. Thus, in order to maximize an LHTES system’s performance, these elements should be addressed concurrently. According to a study in the literature, PCM has many shortcomings, including poor specific heat and thermal conductivity [6,7], phase separation [8], corrosion potential [9], leakage problems [10], and supercooling [11]. As a consequence, much work has been devoted to creating a variety of methods for enhancing PCMs, including shape-stabilized PCM [12,13], additives [14], and micro- or nano-encapsulation [15,16].

Additionally, the performance of PCMs may be improved by improving their container’s design and operating characteristics to allow faster charging and discharging through improved heat transmission. To achieve high solar energy absorption, heat storage, and thermal conductivity, Zhang et al. [17] designed shape-stabilized phase change composite materials, including a support matrix (copper foam), surface modifiers (reduced graphene oxide and graphene oxide), and organic PCM (paraffin and PEG10000). The results indicate that copper foam modified with carbon materials offers a significant number of active sites for the PCM to attach to, which are stable and unlikely to leak. When compared to the pure PCM, the thermal conductivity of the new PCM composite is boosted by 300 percent.

Numerous studies [18,19,20,21] have proven, for example, that raising the mass flow rate improves the overall heat transfer coefficient and increasing the temperature of the thermal fluid at the input raises the temperature difference. As a consequence of increasing both of these parameters, the melting rate and stored energy rise [22]. PCM melts primarily because of natural convection. Consequently, numerous researchers have experimented with different PCM container shapes and orientations in an attempt to increase convection currents and, hence, minimize the melting time [23,24,25]. Mehryan et al. [26] theoretically examined the melting process of a PCM within an inclined compound enclosure, partly filled with a porous medium using a deformed mesh method. The results indicate that when the thickness of the porous layer rises, the melting and heat transmission rates increase. The melting rate is greatest when the enclosure is angled 45°. Iasiello et al. [27] presented experimental and numerical studies on PCMs coupled with aluminum foams under different orientations, number of pores per inch (PPIs), heat fluxes, and porosities. The findings indicate that decreasing the porosity significantly lowers the melting time, while PPI has little impact and only minor changes may be found due to orientation variation. Pu et al. [28] investigated how to increase the melting rate in a PCM-based shell-and-tube thermal energy storage unit by utilizing multiple PCMs with gradient copper foam. The simulation findings indicated that multiple radial PCMs had no thermal storage benefit over single PCMs. Copper foam’s negative gradient provides the most efficient heat transmission. Esapour et al. [29] investigated the solidification and melting rates of RT 35 PCM embedded in porous copper metal foam in a multi-tube horizontal heat exchanger system for energy storage. Mahdi et al. [30] discussed enhancing heat transmission in a PCM-based shell-and-tube TES unit by using cascaded metal foam. The study’s findings indicate that the PCM/metal foam composite foam cascading may significantly reduce energy storage and recovery durations. Zhao et al. [31] investigated the possibility of enhancing the heat transfer capacity of PCM in low- and high-temperature thermal energy storage systems by utilizing open-cell metal foams. The findings show that metal foams perform well in terms of heat transmission, owing to their continuous interconnected structures. They may, however, mitigate the effects of free convection in the PCM’s liquid zone, especially for PCMs with low viscosities. This means that metal foams may not always improve heat transmission under all conditions.

Fins are well known as an efficient means of enhancing heat transfer rate. Many types of fins have been utilized to increase the heat transfer rate in PCM, including longitudinal, radial, or circular fins and pin fins [23,32]. Suraparaju et al. [33] examined the yield improvement potential in a solar still they developed using a single basin and a solid staggered pin finned absorber placed into a paraffin wax bed. To further advance in the freshwater productivity from a solar still, another study [30] created a new bottom-finned (solid and hollow) absorber basin to improve heat transmission between the absorber and the PCM. They reported that the solar still with a hollow-finned absorber placed into the energy storage is more effective than others and is economically feasible for producing drinkable water. Abu-Hamdeh et al. [34] conducted a sensitivity analysis to ascertain the effect of PCM thermophysical characteristics on the utility of PCM-based heat sinks. They reported that raising the melting temperature decreased the efficacy of the PCM-filled heat sink. Other research indicates that increasing the thermal conductivity of PCMs via various techniques significantly improves heat transfer in PCMs. For example, the addition of nanoparticles to PCMs resulted in developing a new class of PCMs, dubbed NEPCM (nanoparticle-enhanced phase change materials), with increased thermal conductivity. As a result, the melting and solidification rates of PCM rise [35]. This effect may be amplified further by raising the nanoparticle dosage. However, this addition increases the viscosity in the PCM, impeding natural convection. As a result, the concentration of nanoparticles must be managed. Laouer et al. [36] examined the effect of a magnetic field and nanoparticle concentration on the melting of Cu-Ice NEPCM in a rectangular cavity at fluctuating temperatures. The findings indicated that increasing the Ha significantly prolongs the melting time at high Ra. Additionally, the inclusion of nanoparticles leads to a significant reduction in melting time at low Ra. However, it has a detrimental impact on the melting time at high Ra in the presence of a magnetic field as it prolongs the melting time in these conditions by 7%. Zhuang et al. [37] investigated the process by which different NEPCMs transition between non-Newtonian and Newtonian rheological regimes throughout the melting process, from macro and micro viewpoints. The findings show that adding nanoparticles to pure paraffin may boost its thermal conductivity. At low shear rates, all of the nanofluids examined function as shear-thinning fluids, with the exception of the NEPCM containing 1% Al_2_O_3_. All nanofluids exhibit Newtonian behavior at high shear rates. Kumar et al. [38] conducted an experimental study into the heat transfer characteristics of palmitic acid embedded with cupric oxide nanoparticles at various mass fractions, ranging from 0.3 to 0.8%. Sheikholeslami et al. [39] evaluated the performance improvement in CuO nanoparticles with various shapes when dispersed in water employed as a PCM in a complex-shaped energy storage system. The results suggested that using nanoparticles accelerated the charging process and that platelet-shaped nanoparticles provide the fastest pace. More recently published papers, those considering using NePCM for enhancing thermal performance of TES units, can be found in [40,41,42,43].

The current study explores the complete melting process of paraffin wax PCM enhanced with copper nanoparticles within a shell-and-tube TES unit. Given the critical role of the PCM’s container and fins in defining the heat transfer process in TES, it is vital to compare the effect of the TES structure on thermal efficiency. The impacts of nanoparticle volume fraction, fins, and TES unit designs on the thermal performance of the TES system are specifically explored. The following summarizes the contents of this article: Section 2 presents the physical model, which includes the design of geometry and the characterization of the thermal properties of PCMs and nanoparticles, followed by Section 3, where the employed mathematical approach is described. The study findings are discussed in Section 4, while the conclusions are given in Section 5.

## 2. Problem Description and Formulation

### 2.1. Physical Model

**Figure 1:**
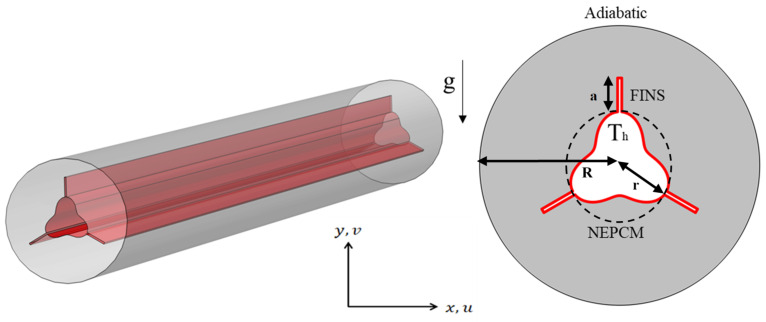
A 3D view of the tube-and-shell TES and a two-dimensional illustration of the studied model with boundary conditions.

The physical model of the shell–tube systems used in this study is identical with the common shell-tube heat exchanger found in the industry. However, for the purpose of attempting to enhance the thermal performance of the system, the heat exchanger tube was redesigned into a trefoil-shaped finned cylinder, as shown in Figure 1, instead of a simple cylindrical tube. Nanoparticles are added to the PCM to increase heat transmission between the heat transfer fluid (HTF), flowing down the tube and the PCM. Given the extensive lengths of shell-and-tube LHTES systems, this system’s cross-section is explored to reduce computational cost and time. PCM is defined in this paper as nano-enhanced paraffin wax. The inner and outside diameters of the annuli PCM container are set to 15 mm (2 × r) and 40 mm (2 × R), respectively. The thermophysical characteristics of paraffin wax and copper nanoparticles are presented in Table 1.

**Figure 2:**
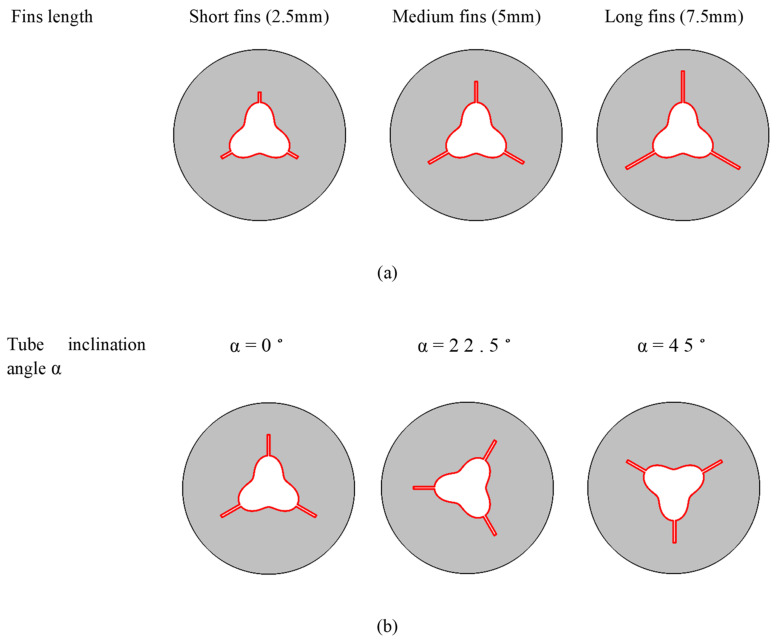
Introduction of the studied cases in the present article for (**a**) various fin lengths and for (**b**) various tube inclination angles α.

NePCM would have the same phase as PCM and experimental data show that nanoparticle addition affects the thermophysical characteristics of PCM. In this work, copper nanoparticles having a diameter of 50 nm and volume percentages of 0 (pure PCM), 4%, and 8% are employed since Xuan et al. [44] reported that nanoparticles with a diameter of less than 100 nm form a homogenous flow with the base fluid. Fins enlarge the contact surface between the PCM and the inner tube. Three cases are explored for the undulating inner cylinder tilt angle (α = 0°, 22.5°, and 45°). Furthermore, three fin lengths (a = 2.5, 5, 7.5 mm) are examined. Figure 2 illustrates the various studied cases.

**Table 1:** Thermophysical properties of the PCM and nanoparticles [45,46].

Property	PCM (Liquid)	PCM (Solid)	Nanoparticles
Density (kg/m^3^)	775	833.6	8954
Specific heat (kJ/kg K)	2.44	2.384	0.383
Thermal conductivity (W/m K)	0.15	0.39	400
Melting temperature (K)	327.15		-
Kinematic viscosity (m^2^/s)	8.31 × 10^−5^		-
Latent heat of fusion (kJ/kg)	184.48		-
Thermal expansion coefficient (K−1)	7.14 × 10^−3^		67 × 10^−5^

### 2.2. Initial and Boundary Conditions

The boundary conditions used in the simulation are depicted in Figure 1. At the internal surface of the shell and the external surface of the HTF tube, a no-slip boundary condition is imposed. The HTF tube’s surface always retains its temperature at T_h_ = 333 K. The temperature of the PCM is initialized to 323 K. The surface of the shell is assumed to be adiabatic.

## 3. Mathematical Model

Roughly speaking, in engineering and applied sciences, mathematical modeling, analysis, and techniques are widely used (see for instance [47,48,49,50,51]). With that has been said, an enthalpy–porosity analysis is a well-used approach for assessing unstable heat transfer situations, such as PCM phase transitions. The enthalpy–porosity approach is used to simulate the melting and heat transfer properties of NePCM embedded fins. The advantage of the enthalpy–porosity technique is that it does not need direct monitoring of the phase contact; rather, it derives the energy equation by employing enthalpy and temperature over the whole calculation domain. Due to the phase transition process’s significant nonlinearity, its challenges grow more complicated. To simplify the calculation, the following parameters are considered:

- The flow of liquid NePCM is regarded to be incompressible and laminar.
- Because PCM’s physical characteristics stay largely constant during the phase shift, they may be considered constant.
- Ignoring the volumetric effect of viscous dissipation and sources of heat.
- Based on the above assumptions, the governing equation for the melting of NePCM in the wavy finned enclosure may be derived. The equations may be written as follows [47].

Continuity equation:

(1) $$\nabla \cdot \left(\vec{V}\right)=0$$

Momentum equation

(2) $$\frac{\partial \left(\rho_{np}\vec{V}\right)}{\partial t}+\nabla \cdot \left(\rho_{np}\vec{V}\right)=-\nabla P+\mu_{np}\nabla^{2}\vec{V}-S_{b}+S_{a}$$

where the subscript *np* refers to the nano-enhanced PCM/$S_{a}$ a is the source term for the porosity function

(3) $$S_{a}\;\mathrm{define}\;\mathrm{as}\;S_{a}=-A\vec{V}\;\mathrm{with}\;A=\frac{{\left(1-\eta \right)}^{2}}{\left(\pi^{3}+10^{-3}\right)}C$$

and

(4) $$\nabla \left(P\right)=-\frac{{\left(1-\eta \right)}^{2}}{\eta^{3}}C\cdot \vec{V}$$

$S_{b}$ is the Boussinesq approximation to the buoyancy force; the value is as follows

(5) $$S_{b}={\left(\rho_{\;}\beta \right)}_{np}\left(T-T_{m}\right)\vec{g}$$

The vector of fluid velocity is denoted by $\vec{V}$. The two-dimensional model specifies the axial and radial velocity vector components as follows:

(6) $$V_{axial\;}=v\;and\;V_{radial\;}=u\;$$

Energy equation

(7) $$\frac{\partial \left(H\right)}{\partial t}+\nabla \cdot \left(\vec{V}H\right)=\nabla \cdot \left(k_{np}\nabla T\right)$$

where *H* signifies a certain enthalpy and is stated in the following manner:

(8) H=h+ΔH

where *h* is the sensible enthalpy denoted by the formula:

(9) $$h=h_{ref}+\int_{T_{ref}}^{T}{\left(\rho c_{p}\right)}_{np}dT$$

the value of C is taken as $C=10^{6}$.

Additionally, η is the equation for the liquid portion of the liquid/solid zone, which assists in defining the zone of calculated cells, where the liquid zone equals η=1 and the solid zone equals η=0. In contrast, the mushy zone equals 0<η<1, and can be expressed as follows:

(10) $$\eta \left(T\right)=\left\{\begin{array}{c} 0\;if\;T<T_{S}\;\\ 1\;if\;T>T_{l}\\ \;\frac{T-T_{S}}{T_{l}-T_{S}}\;if\;T_{l}>T\geq T_{S}\; \end{array}\right\}$$

with $T_{l}$ and $T_{S}$ denoting the NePCM’s liquid and solid temperatures, respectively. It is possible to represent the liquid fraction expression as flows:

(11) $$\eta \left(T\right)=\left\{\begin{array}{c} 0\;if\;T<\left(T_{m}-\Delta T\right)\;\\ 1\;if\;T>\left(T_{m}+\Delta T\right)\\ \;\frac{\left(T-T_{m}+\Delta T\right)}{2\Delta T}\;if\;\left(T_{m}+\Delta T\right)>T\geq \left(T_{m}-\Delta T\right)\; \end{array}\right\}$$

In the case of pure PCM, the parameters are calculated using the thermophysical properties of paraffin wax; in the case of NePCM, the parameters are estimated using a combination of paraffin wax and copper nanoparticle properties, as indicated in Table 1. The accompanying equations use generic notations for the thermophysical properties of PCM and NePCM. The following density and specific heat capacity values are computed for the nano-PCM material:

The density and specific heat capacity of the nano-PCM material are determined using the following equations:

(12) $$\rho_{np}=\left(1-\varphi \right)\rho_{p}+\varphi \rho_{n}$$

(13) $${\left(\rho c_{p}\right)}_{np}=\left(1-\varphi \right){\left(\rho c_{p}\right)}_{p}+\varphi {\left(\rho c_{p}\right)}_{n}$$

where *n* and *p* are corresponding subscripts for nanoparticles and PCM.

The volumetric fraction of nanoparticles added to the PCM is indicated by φ.

Using the following set of equations, one can determine the latent heat of fusion, the effective thermal conductivity, and the thermal expansion coefficient of NEPCM.

(14) $${\left(\rho L\right)}_{np}=\left(1-\varphi \right){\left(\rho L\right)}_{p}$$

(15) $$k_{np}=\frac{k_{n}+2k_{p}-2\varphi \left(k_{p}-k_{n}\right)}{k_{n}+2k_{p}+\varphi \left(k_{p}-k_{n}\right)}k_{p}$$

(16) $${\left(\rho \beta \right)}_{np}=\left(1-\varphi \right){\left(\rho \beta \right)}_{p}+\varphi {\left(\rho \beta \right)}_{n}$$

The entropy created as a result of thermal irreversibility (heat transfer) and entropy created as a result of the flow’s irreversibility (presence of a friction factor) are respectively, equal to

(17) $$S_{ht}=\frac{k_{nf}}{{\bar{T}}^{2}}\left[{\left(\frac{\partial \bar{T}}{\partial x}\right)}^{2}+{\left(\frac{\partial \bar{T}}{\partial y}\right)}^{2}\right]$$

(18) $$S_{f}=\frac{\mu_{nf}}{\bar{T}}\left\{2\left[{\left(\frac{\partial \bar{u}}{\partial x}\right)}^{2}+{\left(\frac{\partial \bar{v}}{\partial y}\right)}^{2}\right]+{\left(\frac{\partial \bar{u}}{\partial x}+\frac{\partial \bar{v}}{\partial y}\right)}^{2}\right\}$$

The total entropy, which includes the increase in entropy due to heat transmission and fluid friction, and the Bejan number, which is the ratio of irreversible heat transfer to total entropy, are calculated using the following formulas.

(19) $$S_{tot}=S_{ht}+S_{f}$$

(20) $$Be=\frac{S_{ht}}{S_{tot}}$$

**Figure 3:**
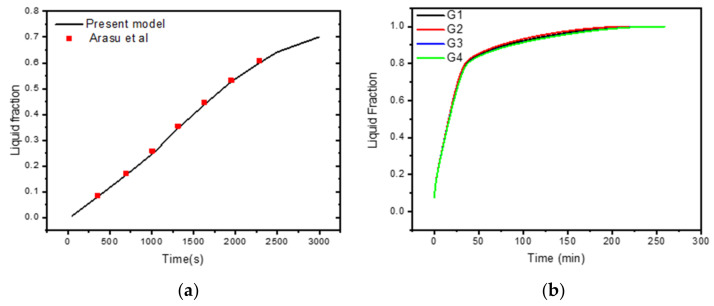
(**a**) Comparison of numerical results with [52]; (**b**) grid independent study.

The mesh independence analysis is conducted by comparing the average liquid percentage over time for the different grid sizes listed in Table 2. Figure 3a illustrates a computational grid, in which the mesh must be carefully calibrated across the domain to account for the melting interface’s movement at each time step. Figure 3b illustrates the effect of various mesh sizes on the liquid fraction during the melting process. Based on the results of the mesh independence analysis (shown in Figure 3b), mesh G2 is used to perform all numerical simulations in this research.

## 4. Results and Discussion

A trefoil-shaped tube is designed to melt paraffin wax phase change material (NePCM) incorporated with nanoparticles (Cu), which is put annularly between the tube and external shell. The tube is equipped with three fins at the end sides of the trefoil-shaped tube. The NePCM shell serves as an energy storage unit. In this section, the concentration of nanoparticles, the fin length, and the finned tube orientation (inclination angle) are investigated against the temperature, liquid fraction, Bejan number (the contribution of heat losses in the total entropy generation), and Nusselt number to represent heat transfer and melting process.

### 4.1. Nanoparticle Concentration

**Figure 4:**
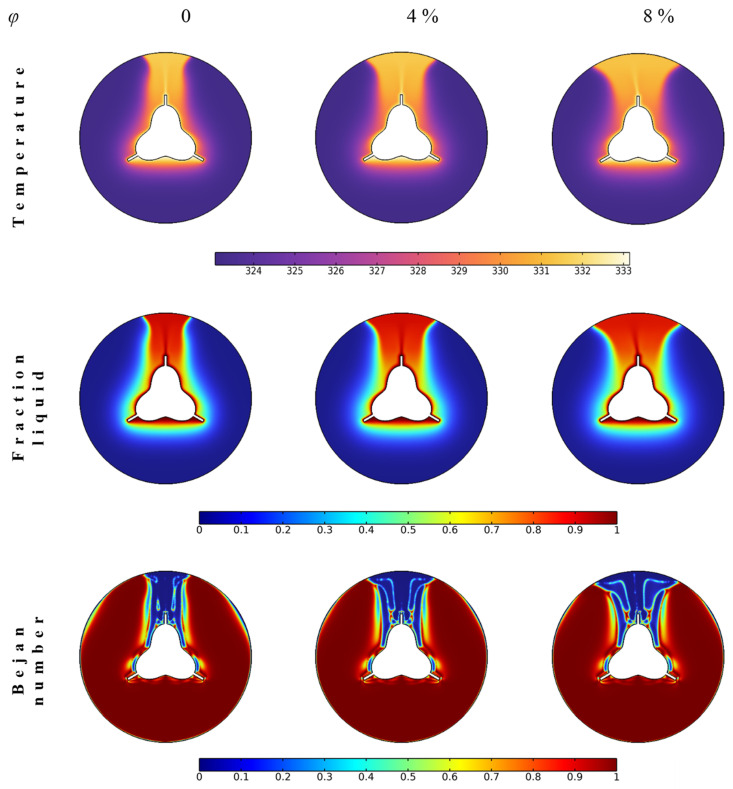
The contours of temperature, liquid fraction, and Bejan number for different cases of nanoparticle concentrations after 20 min.

The nanoparticles are added to enhance the thermal characteristics of PCM, such as thermal conductivity, so that heat transfer rates and melting process are expected to be improved. Three concentration values are considered, i.e., 0, 4, and 8 vol%. It is obvious from Figure 4 that the melting process covers larger regions of the shell unit for the highest concentration case. The temperature contours show higher values distributed between the middle and top of the shell. That is because the heat transfer is controlled by free convection heat transfer influenced by the buoyancy effect. The liquid fraction is also higher in this region. The NePCM is heated by the finned tube and lower-density melted particles rise. The melting process propagates to other shell regions due to the conduction heat transfer from the fins and the convection heat transfer from the liquid to the solid–liquid interfaces. The melted regions have higher temperatures closer to the finned tube temperature, resulting in lower heat transfer. Thus, the heat losses are smaller, which is also confirmed by the Bejan number values in the upper region. In such places, the contribution of liquid flow friction is significant. In contrast, the rest of the shell unit shows a higher Bejan number due to higher heat transfer rates and a continuous melting process. Figure 4 concludes that higher nanoparticle concentrations lead to expediting the melting process.

### 4.2. Melting Time

**Figure 5:**
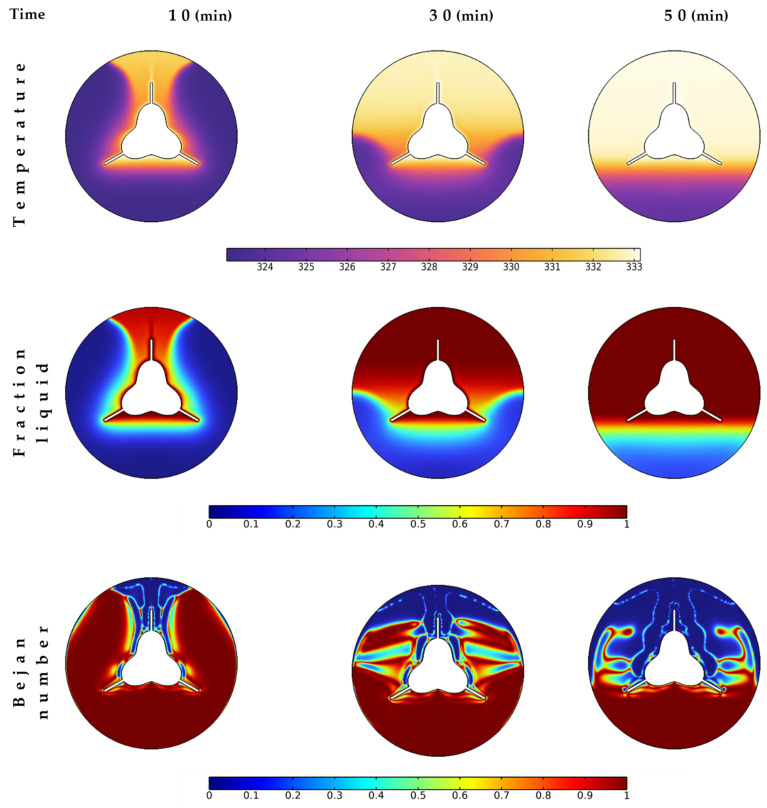
The contours of temperature, liquid fraction, and Bejan number during different melting times with nanoparticle concentration of 4%.

The melting time is of prime importance in thermal energy storage applications. When a high quantity of NePCM melts quickly, more energy can be stored. In this section, three melting times (i.e., 10, 30, and 50 min) are investigated to check the melting process of the investigated NePCM, as shown in Figure 5. Almost 30% of the PCM is melted in the first 10 min and 60% melts for 30 min. At 50 min, about 77% of the material is melted from the top and middle regions. The heat transfer to the bottom region is due to conduction; thus, it is expected to take longer to obtain a fully melted unit.

The temperature contours show higher temperatures for the melted PCM region that reaches closer to the tube temperature (~333 K). In comparison, at the bottom, the temperature is still lower than 326 K for all cases. The higher liquid fraction values take similar places to the hot regions shown in the temperature contours. Fully melted regions (liquid phase of PCM) are found first at the top of the shell unit and propagate with time passing (under applied heating process from the finned tube surface). As fewer heat transfer rates exist in the hot regions, the Bejan number is very low, announcing the flow friction responsibility for the majority of the entropy generation. At the same time, the heat losses are still at the bottom of the shell unit due to the continuity of the melting process.

### 4.3. Fin Length

**Figure 6:**
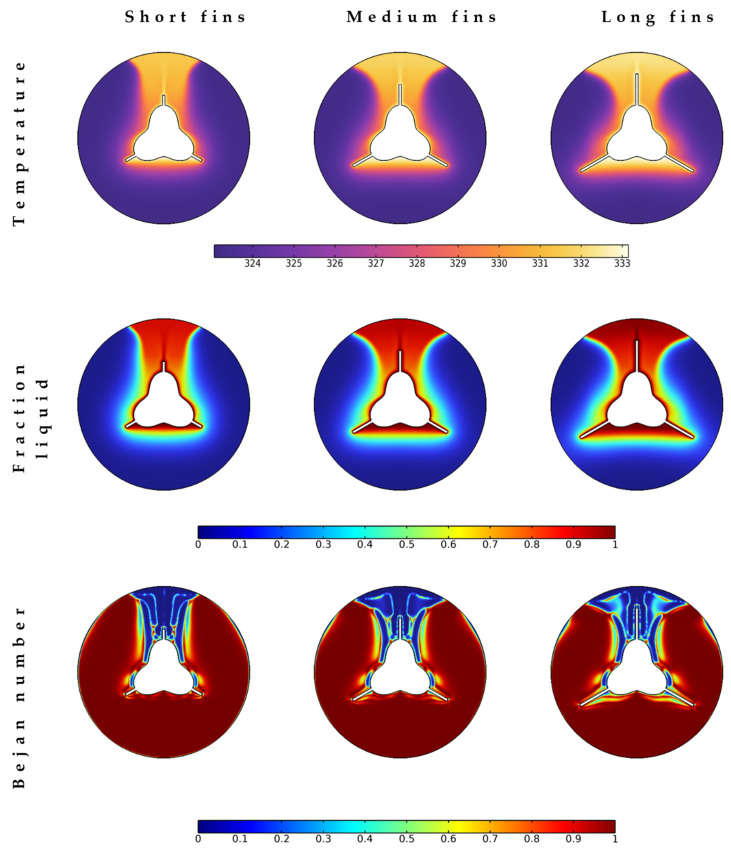
The temperature, liquid fraction, and Bejan number contours at different fin lengths after 20 min.

Because heat transfer mechanisms are conductive and free convective, the fins’ presence substantially assists the increased heat transfer rates and enhanced melting process. Th equipped fins are investigated for three different lengths: low, medium, and long, as shown in Figure 6. A look at the temperature and fluid friction contours provides the fact that long fins are the best to expedite the melting process and quickly secure the required latent heat through heat transfer from the tube to the melting regions. The longer fins have a larger hot liquid region, so the Bejan number has the lowest values in these regions. Increasing the length of fins provides more heat transfer for places away from the tube. It is also expected that the heat transfer could be further improved if the fins are extended to the external wall.

### 4.4. Inclination Angle

**Figure 7:**
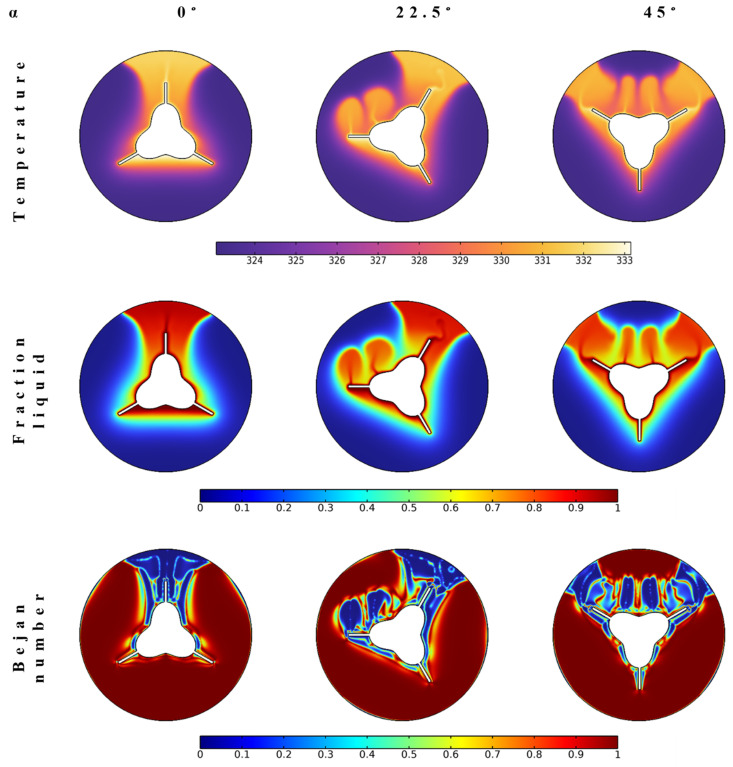
The temperature, liquid fraction, and Bejan number contours at different tube fins after 20 min.

As the length of the fins is important, the location of these fins inside the PCM is also important because heat transfer is affected by the buoyancy effect. Three orientations by rotating the finned tube are considered, i.e., 0, 22.5, and 45°. The 0° inclination means that one fin is vertical at the top and the other two fins are inclined between the middle and bottom of the shell unit. In contrast, the inclination of 45° has the opposite direction to that of 0°, in which the vertical fin is at the bottom of the shell unit. Figure 7 shows that the temperature and fluid fraction values are better for the fins’ inclination of 45°, which shows the bottom fins immersed down vertically to be closer to the bottom shell wall. Thus, the heat transfer by free convection can start from the bottom regions.

Moreover, the two fins between the top and middle of the unit spread wider, making the heat transfer at the top broader. Because the heat transfer and melting process is better for the case of 45° inclination, the Bejan number has lower values in these hot regions, demonstrating low heat losses. The case of 22.5° inclination also reveals good heat transfer, but it comes after the 45° inclination case.

### 4.5. Temporal Performance of the Tube–Shell Unit

**Figure 8:**
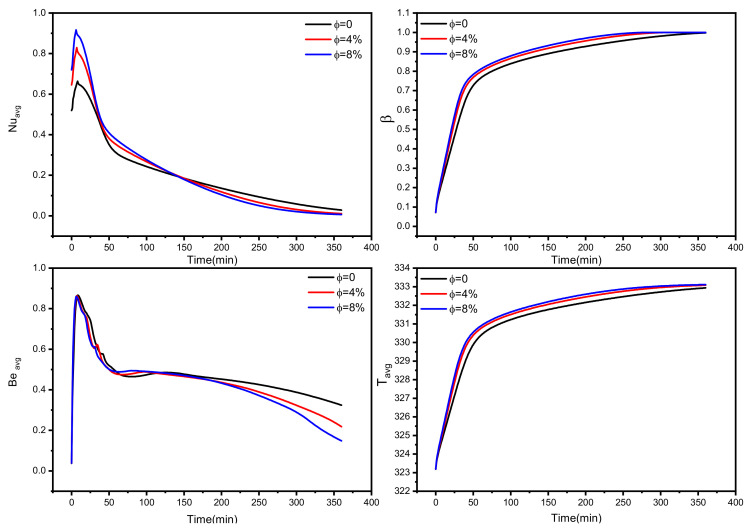
The temporal effect of nanoparticle concentration on the investigated tube-shell TES performance is the average Nusselt number, liquid fraction, average Bejan number, and average PCM temperature.

Figure 8 depicts the history profile of the average Nusselt number (Nu_avg_), the liquid fraction (β), average Bejan number (Be_avg_), and average PCM temperature (T_avg_) as a function of nanoparticle concentrations. As discussed in Figure 4, the higher concentration results in better heat transfer performance. This is also confirmed in Figure 8 in the first 150 min, in which about 90% of the unit is melted, influenced by higher heat transfer rates, as shown by higher Nu_avg_ values (see the figure at the top-left side) and lower average Bejan number values (see the figure at the bottom-left side). The liquid fraction and temperature values are also higher for this case (8 vol% nanoparticles) during the investigated time (360 min). The values of Nu_avg_ are lower for the case of 8 vol% nanoparticles after 150 min, in which most of PCM is melted and not much heat transfer exists, compared to the other two cases that still have a significant melting process. Another important remark that can be concluded from Figure 8 is that about 55 min is sufficient to melt 80% of the PCM while the rest needs >200 min to be fully melted. This is pertinent to the bottom region, where the buoyancy effect is low and far away from the fins. About 99% of the PCM is melted over 240 min for the 8 vol% case, while that takes 325 min for no nanoparticle inclusion (0 vol%). About 27% enhancement in melting time reduction is evaluated for incorporating nanoparticles with 8 vol%.

**Figure 9:**
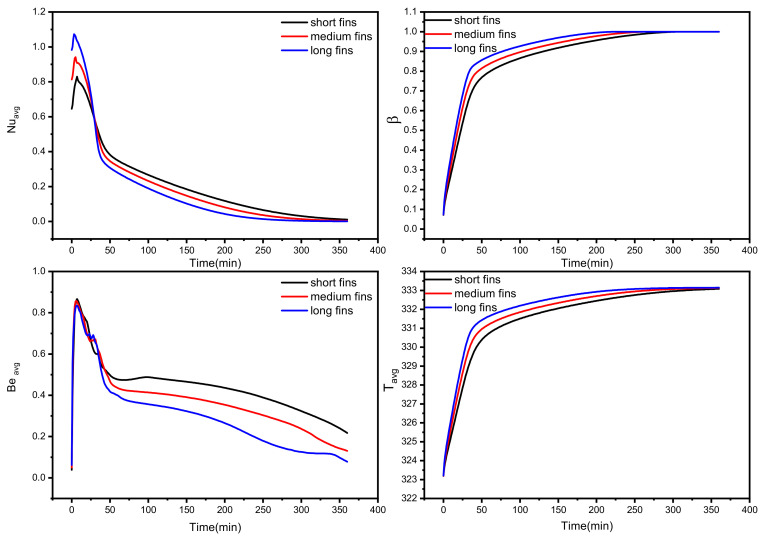
Temporal effect of fin length on the investigated tube–shell TES performance in terms of average Nusselt number, liquid fraction, average Bejan number, and average PCM temperature.

The temporal effect of the fin length on the melting process and heat transfer performance is displayed in Figure 9. As evident, 30 min is sufficient to melt about 76% of the PCM using long fins (with higher values of Nu_avg_), whereas the short fins need around 50 min (40% higher time than the long fins) to have the same melting ratio. The long fins also showed better liquid fraction and average temperature values and lower Be_avg_ values during the investigated time. Thus, long fins are recommended.

**Figure 10:**
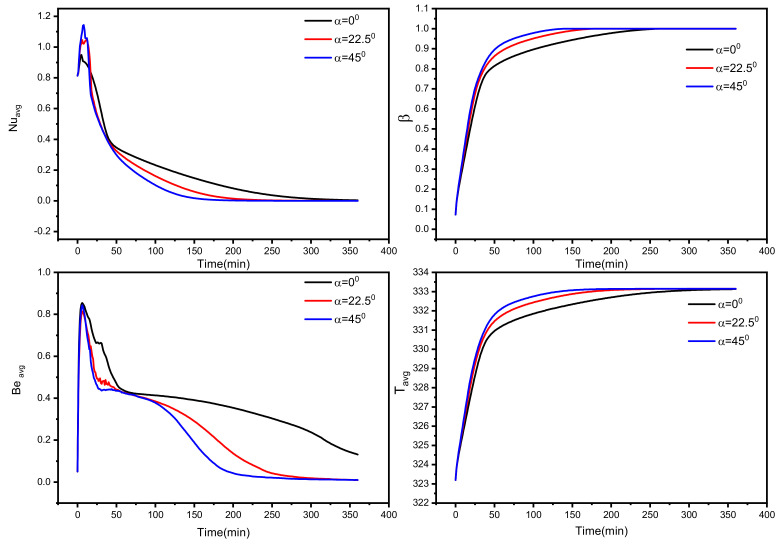
Temporal effect of tube position (inclination angle) on the investigated tube–shell TES performance in terms of average Nusselt number, liquid fraction, average Bejan number, and average PCM temperature.

Figure 10 demonstrates the importance of the fins’ orientation inside the TES unit at three inclination angles, 0, 22.5, and 45°. This figure considers the medium-length fins. The temporal effect of the average Nusselt number, liquid fraction, average Bejan number, and average PCM temperature shows good performance with the finned tube inclination of 45° (which has one vertical fin that heats the bottom of the TES unit). It could be seen that the melting ratio reaches 99% for 115 min using the finned tube inclination of 45°, while it takes 228 min for the no-inclination case (α = 0°). About 50% of melting expedition can be obtained by just having the proper orientation for the fins. Since the buoyancy effect is important, expanding its influence to start from the bottom of the shell unit results in enhanced heat transfer rates and melting process. Using more than three long fins with this position (45°) is also expected to eliminate the melting time to be less than 110 min.

## 5. Conclusions

This study uses a trefoil-shaped tube to heat paraffin wax (NePCM) incorporated with nanoparticles (Cu), placing it in a cylindrical shell. The shell serves as a thermal energy storage unit. The tube is equipped with three fins to enhance the heat transfer and NePCM melting process. The influential parameters considered are the concentration of nanoparticles, the fin length, and the finned tube orientation (inclination angle). At the same time, the results are represented by PCM temperature, liquid fraction, Bejan number, and Nusselt number. For the input parameters used in this study, the following remarks are derived:

- Incorporating Cu nanoparticles with PCM materials enhances the thermal and melting performance and minimizes the entropy generation. The best results (with a 27% reduction in melting time) are found to be with the use of 8 vol% of nanoparticle concentration.
- Using long fins could enhance the heat transfer characteristics and reduce the melting time by about 40%.
- The orientation of the fins is crucial since they reach the bottom regions of the TES unit to benefit from the buoyancy effect. The position of the fins for 45° inclination could achieve a 50% expedition in the melting process.
- Based on the mentioned remarks, incorporating nanoparticles with PCM and using long fins in a proper position lead to an enhanced PCM melting process for the thermal energy storage units.

## Figures and Tables

**Table 2:** Numerical test results for grid independency study.

Mesh	G1	G2	G3	G4
**Number of Elements**	25,496	30,772	58,662	81,967

## Data Availability

Not applicable.
